# Quantifying Geographic Atrophy in Age-Related Macular Degeneration: A Comparative Analysis Across 12 Deep Learning Models

**DOI:** 10.1167/iovs.65.8.42

**Published:** 2024-07-24

**Authors:** Apoorva Safai, Colin Froines, Robert Slater, Rachel E. Linderman, Jacob Bogost, Caleb Pacheco, Rickie Voland, Jeong Pak, Pallavi Tiwari, Roomasa Channa, Amitha Domalpally

**Affiliations:** 1 A-EYE Research Unit, Dept of Ophthalmology and Visual Sciences, University of Wisconsin, Madison, Wisconsin, United States; 2 Depts of Radiology and Biomedical Engineering, University of Wisconsin, Madison, Wisconsin, United States; 3 Wisconsin Reading Center, Dept of Ophthalmology and Visual Sciences, University of Wisconsin, Madison, Wisconsin, United States

**Keywords:** geographic atrophy, deep learning, AI architecture

## Abstract

**Purpose:**

AI algorithms have shown impressive performance in segmenting geographic atrophy (GA) from fundus autofluorescence (FAF) images. However, selection of artificial intelligence (AI) architecture is an important variable in model development. Here, we explore 12 distinct AI architecture combinations to determine the most effective approach for GA segmentation.

**Methods:**

We investigated various AI architectures, each with distinct combinations of encoders and decoders. The architectures included three decoders—FPN (Feature Pyramid Network), UNet, and PSPNet (Pyramid Scene Parsing Network)—and serve as the foundation framework for segmentation task. Encoders including EfficientNet, ResNet (Residual Networks), VGG (Visual Geometry Group) and Mix Vision Transformer (mViT) have a role in extracting optimum latent features for accurate GA segmentation. Performance was measured through comparison of GA areas between human and AI predictions and Dice Coefficient (DC).

**Results:**

The training dataset included 601 FAF images from AREDS2 study and validation included 156 FAF images from the GlaxoSmithKline study. The mean absolute difference between grader measured and AI predicted areas ranged from −0.08 (95% CI = −1.35, 1.19) to 0.73 mm^2^ (95% CI = −5.75,4.29) and DC between 0.884–0.993. The best-performing models were UNet and FPN frameworks with mViT, and the least-performing models were PSPNet framework.

**Conclusions:**

The choice of AI architecture impacts GA segmentation performance. Vision transformers with FPN and UNet architectures demonstrate stronger suitability for this task compared to Convolutional Neural Network– and PSPNet-based models. Selecting an AI architecture must be tailored to the specific goals of the project, and developers should consider which architecture is ideal for their project.

In recent years, the field of artificial intelligence (AI) has proven highly effective in medical image analysis. Typically, AI model development is a multistep process, starting with selection of appropriate AI architecture, curation of training and testing datasets, and fine tuning of hyper parameters. The model architecture serves as the foundation, determining its ability to capture fine details from the images. A variety of architectures are available, each with its own unique strengths and characteristics.^1^ Segmentation AI architecture consists of a framework within which encoder-decoder combinations are configured. The encoders are responsible for extracting relevant information from images, and decoders generate meaningful output based on the extracted features. The choice of encoder and decoder combinations and their organization in a framework forms the basis of the model architecture. The method of decoding has come to take on the name of the entire model or architecture, and the encoder has become a swappable sub-piece of the overall architecture. Other variables, called *hyperparameters*, including learning rate, batch size, and number of layers of the model also need tuning to achieve a balance between model's performance and computational cost. This process ensures that the model can generalize effectively on unseen data, a critical requirement for real world applications.

Age-related macular degeneration (AMD) is a leading cause of vision impairment worldwide, with geographic atrophy (GA) being a hallmark of its advanced stage.^2^ GA is characterized by the progressive loss of retinal pigment epithelium (RPE) cells and photoreceptors, resulting in irreversible vision impairment.^3^ Accurate measurement of GA is crucial for disease assessment and monitoring, as well as for developing and evaluating potential treatments. Fundus autofluorescence (FAF) imaging using blue light autofluorescence has emerged as the preferred modality for imaging GA as it provides high contrast grey scale images where atrophy presents as dark (hypo autofluorescence) lesions with distinct boundaries.^4^ Enlargement of GA area FAF images is an important outcome for clinical trials.^5^ Traditional manual measurements are time-consuming and subject to inter- and intra-observer variability.^6^ Additionally, these measurements are typically performed in reading centers using methods not amenable within the busy clinic workflow.

Segmentation of GA using deep learning models has been generally successful.^7^^–^^11^ However, owing to the variable phenotypes and manifestations of GA, accurate identification of the hypo-autofluorescence boundary and generating precise segmentation across datasets is a challenging task.^12^ Selection of proper AI architecture that captures the heterogenous patterns of GA is critical for attaining robust segmentations and better model performance. A comprehensive evaluation of popular segmentation frameworks could act as a guideline for selecting AI architecture that yields high performance for the GA segmentation task. In this study, we explored three different segmentation architectures combined with four widely used encoders leading 12 distinct combinations of AI models to identify the best AI framework for segmentation of GA on FAF images.

## Methods

### Training Dataset: Image Acquisition

Age-Related Eye Disease Study 2 (AREDS2)^13^ was a multicenter randomized clinical trial designed to study the effects of oral supplements on progression to advanced AMD. The study was conducted under institutional review board approval at each site and written informed consent was obtained from all study participants. The research was conducted under the Declaration of Helsinki and complied with the Health Insurance Portability and Accountability Act. Participants at high risk of developing late AMD because of either bilateral large drusen or late AMD in one eye and large drusen in the fellow eye were enrolled. Development of either central GA or neovascular AMD was the primary AREDS2 study outcome.

An autofluorescence ancillary study was initiated to obtain autofluorescence images from a subset of participating clinics (36 of 90 sites) based on availability of imaging equipment.^14^ Sites were permitted to join the ancillary study at any time after imaging equipment became available during the study period between the first AREDS2 visit and five-year follow-up visit (2007–2013). FAF images were obtained from Heidelberg Retinal Angiograph (HRA, Heidelberg, Germany) by certified photographers. A single image was acquired at 30° centered on the macula, captured in high-speed mode (768 × 768 pixels) using the automated real time mean function set at 14. Images were exported as tiff format to the Wisconsin Reading Center (formerly Fundus Photograph Reading Center) for evaluation by certified graders.

For this project, FAF images with GA were included from AREDS2 study visits at year 4, 5 and 6, because that was the time frame where most sites with FAF capabilities joined the ancillary study to maximize the diversity of sites. There were 1501 FAF images corresponding to these visits. A total of 601 FAF images from 362 eyes (271 participants) with GA were considered from the AREDS2 study for training the segmentation models. Of these 200 (55.2%) eyes had only one visit, 162(44.8%) eyes had two or more visits. Cross-validation required split at subject level.

### Training Dataset: Image Labelling

The ground truth labels of GA were manually drawn by three independent graders. Images were split between three independent graders with a random subset undergoing repeat grades for intergrader agreement. Hypo-autofluorescence or GA was classified as well-defined, homogenously black areas with a minimum size of 250 microns in its widest diameter or an area greater than 0.05 mm^2^.^15^ Areas of hypo autofluorescence within the entire macula centered FAF image were demarcated using Photoshop (Adobe Inc. v 24.4.1) with a red outline and filled in with the paint bucket tool. Images were deemed ungradable and excluded from this study if the border of GA merged with peripapillary atrophy and could not be distinguished, if the GA extended outside the field of the image, or if poor image quality prevented clear delineation of GA borders. In Heidelberg FAF images, the macula was assumed to be involved if the hypo-autofluorescent patch merged with the darkness of the macula and there was no clear region demarcating the two. Optical coherence tomography (OCT) images, which provide a more accurate assessment of foveal involvement, were not available. The intergrader agreement on detecting foveal involvement of GA was 84% (kappa 0.07). A 200 µm scale bar is provided on Heidelberg FAF images and was used to calibrate the images. The pixels in red were converted to area measurements in millimeters squared. Areas were summed for eyes with multifocal GA to yield a single value.

### Dataset: External Validation (Testing)

External validation was performed using all screening visit FAF images from a phase 2 study conducted by GlaxoSmithKline (GSK) between 2011 and 2016 (NCT NCT01342926).^16^ This was a multicenter study conducted across 40 centers in United States and Canada and the study concluded that the experimental drug did not slow the enlargement rate of GA compared with placebo. All images were obtained by certified photographers and included 30-degree images centered on the fovea at high speed (768 × 768 resolution) or high resolution (1536 × 1536) and image averaging (ART mean function) set to 25. Inclusion criteria required well-demarcated GA with an area of 1.9 to 17 mm^2^ measured on color fundus photographs of the study eye. For multifocal GA, at least one of the foci had to be ≥1.9 mm^2^ and the total area of GA had to measure ≤17 mm^2^. However, all images submitted for the study irrespective of inclusion criteria range were included in the dataset. Inclusion criteria also required a best corrected visual acuity score of 35 letters or more in the study eye. FAF images were obtained as supplementary images using the same procedures as AREDS2 but were exported to the reading center in the Heidelberg proprietary e2e format. GA segmentation was performed using the same procedures as above in Heidelberg software. Images with annotations were exported in tiff format for AI validation. The external validation dataset consisted of 156 images (156 eyes, 100 participants).

### Comparative Models for Segmentation

We investigated a range of AI models, each with distinct combinations of segmentation architectures and encoder modules. The segmentation architectures included in this study were UNet, FPN (Feature Pyramid Network), and PSPNet (Pyramid Scene Parsing Network), whereas the pretrained encoders consisted of EfficientNet, ResNet (Residual Networks), VGG (Visual Geometry Group), and mViT (Mixed Vision Transformer). This combination resulted in a diverse set of 12 segmentation models.

**Figure 1.**
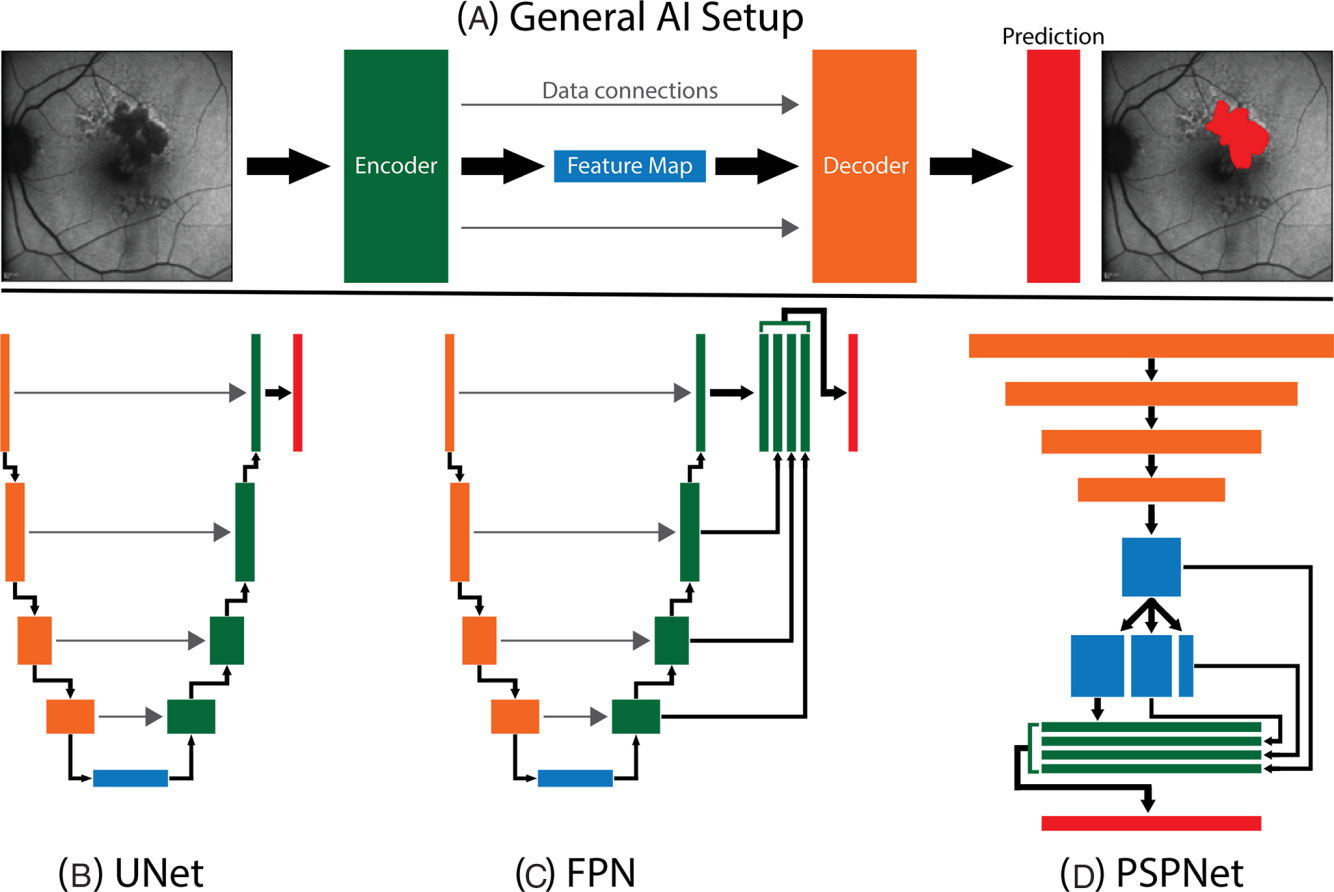
(**A**) A general view of how a segmentation in an AI model is created. An image is passed to an encoder (*orange*), which produces a feature map (*blue*). The feature map is then used as input to a decoder (*green*) to predict a segmentation mask (red). The encoder may send information directly to the decoder (*gray arrows*). (**B**) The general structure and data flow for the UNet architecture. The encoder produced a feature map of reduced height and width dimension which is then upscaled by the decoder to produce a final segmentation mask from the final layer of the decoder. (**C**) The general structure for an FPN. The encoder is unchanged when compared to UNet but the decoder now has extra internal connections that contribute to the final prediction. These additional connections help the decoder with scale or resolution. (**D**) The structure of a PSPNet architecture. The encoder again is mostly unchanged, but the decoder pools the feature map at various sizes and then combines the results to create a final segmentation mask.

We choose the above-mentioned segmentation architectures due to their favorable and distinct attributes of extracting detailed, fine grained and multiscale features for segmentation tasks.^17^ Figure 1 shows a schematic of these architectures. UNet is a U-shaped encoder-decoder model where the input picture/data is compressed into a feature vector (i.e., it shrinks an image down to its key features and then builds it back up to create a detailed map of the image with segmentation).^18^ This helps the model focus on important features by reducing complexity. FPN with its pyramidal structure is an advancement of UNet and makes predictions at multiple resolutions and combines them for an overall prediction.^19^ Last, the PSPNet model uses an innovative approach through its adaptive pooling strategy. It concurrently extracts image feature maps from different regions of the image and then pools them to provide semantic segmentations from the image.^20^ This aggregation of local feature maps helps the model understand both subtle and largescale context from the image.

Among the encoder models used in this study for segmentation, EfficientNet, ResNet and VGG are widely used convolutional neural network (CNN) models, whereas mViT is based on the recently popular transformer model. EfficientNet is an encoder module designed to improve the efficiency of neural networks by balancing and optimizing model dimensions such as depth, width, and resolution.^21^ In segmentation tasks, this optimization is valuable for achieving better performance with fewer resources. ResNet model is composed of residual blocks, containing skip connections which allows direct flow of information across layers, thereby facilitating better training of a deeper model, with large number of layers.^22^ With this residual learning strategy ResNet can effectively capture hierarchical image features for segmentation. VGG model involves stack of convolutional layers, where the kernel size can be varied to extract more intricate features for segmentation task. Last, mViT is a vision transformer, that leverages self-attention mechanisms to capture long-range dependencies and global context in data.^23^ This ability of mViT to understand relationships within the entire image and capture dependencies in spatially distant regions can aid in complex segmentation tasks. Thus each of the CNN and transformer-based encoder-decoder modules possess different attributes that can be beneficial for obtaining precise segmentations on retinal images.

### Training of Segmentation Models

All segmentation models were trained and validated on the AREDS2 dataset and tested on the GSK dataset. A fivefold cross-validation (CV) scheme was implemented on the AREDS2 dataset with each fold containing 120 input images split at subject level. All images were first preprocessed. Images with a Heidelberg label bar had the bar cropped off, resulting in a perfectly square image. The bar was always 100 pixels tall and at the bottom of the picture. Most images were in three-channel RGB format (even though the content was black and white). Those that were single channel were converted to three channels. Images were then resized to dimensions 512 × 512 pixels. The various encoder modules pretrained on ImageNet were incorporated with different designs parameters. For instance, the EfficientNet encoder was scaled at b5 level (parameters = 28 M), ResNet encoder was designed with 101 layers (parameters = 42 M), VGG encoder contained 19 layers (parameters = 20 M) and mViT encoder of type SegFormerB5 (parameters = 81 M). All comparative models were trained in a uniform manner, with a batch size of 4 and learning rate of 0.001, constrained to the available computational resources. We implemented early stopping to prevent models from overfitting by monitoring the validation loss with a patience of 10 epochs. All models were implemented on NVIDIA QUADRO RTX 5000 GPU using Pytorch Lightning and the Segmentation Models Pytorch library. The segmentation performance of all models was evaluated on the external GSK dataset.

### Performance Evaluation of Segmentation Models

#### Statistical Assessment of AI Performance

The segmented GA mask was converted to mm^2^ using known pixel size and the area of segmented GA mask was computed. GA characteristics of the segmented masks were outlined using summary statistics such as area of GA mask. Model performance for each of the 12 models was measured using mean difference, correlation, and dice coefficient between area of segmented GA mask and GA label by human graders. Dice coefficient is measured as the ratio of twice the intersection of the predicted and ground truth regions to the sum of the sizes of the predicted and ground truth regions on an image. A Dice score closer to 1 indicates excellent agreement in spatial overlap of segmented pixels between AI and grader. Dice coefficient was generated for a single internal validation set and for a fivefold CV framework, where the average score from all folds is reported. Additional performance metrics for the segmentation task such as Jaccard index, precision and recall were also computed for all 12 models.^24^ Owing to the variable manifestation of GA, the performance of all models was further specifically evaluated for segmentation of unifocal versus multifocal GA and sub foveal GA subtype.

#### Grader Assessment of AI Performance

**Figure 2.**
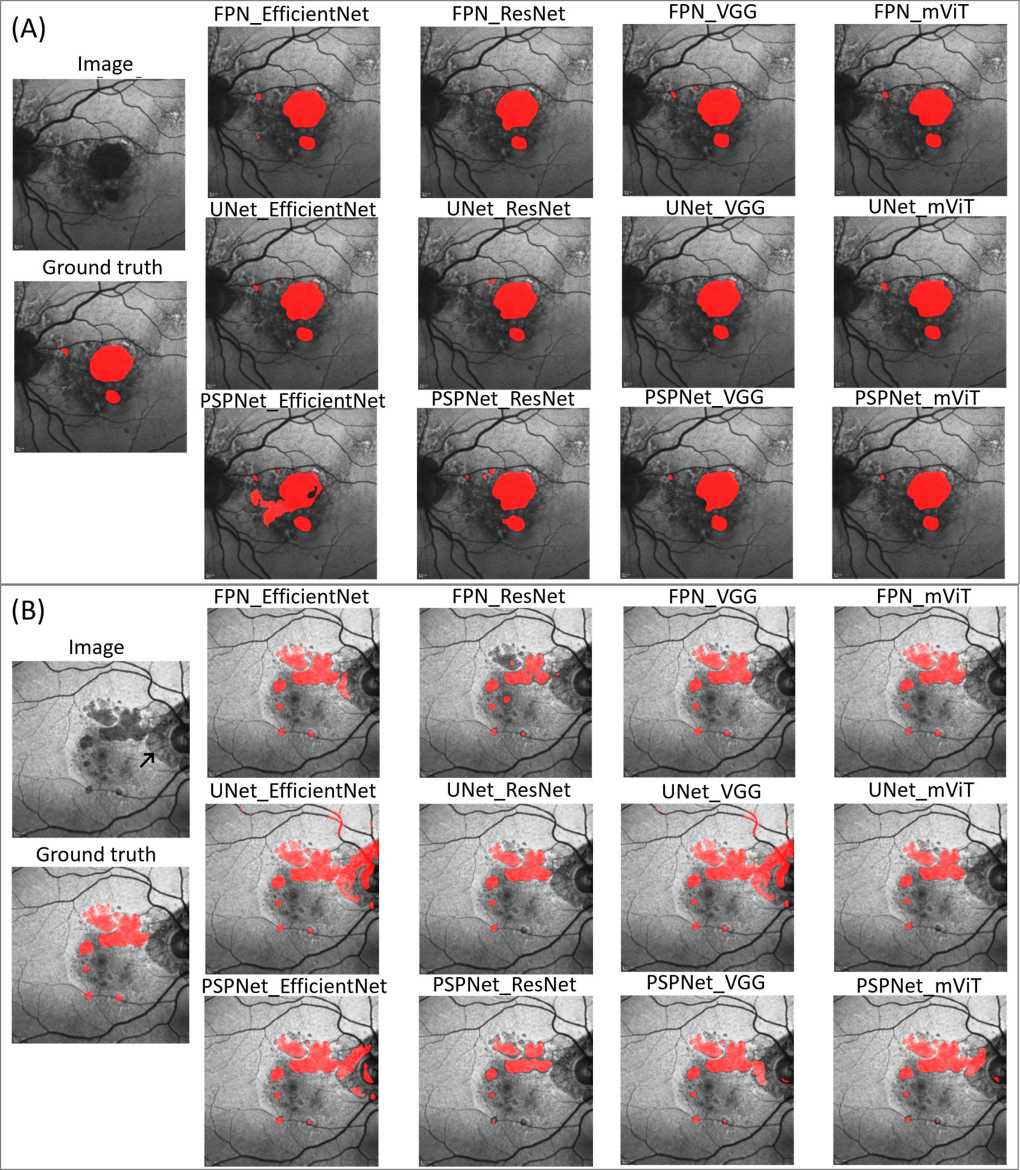
Fundus autofluorescence image with segmentation of geographic atrophy by human grader (treated as the ground truth) and predictions from the 12 AI architectures. (**A**) A multifocal foveal GA that is segmented accurately by UNet and FPN architectures but not by PSPNet. (**B**) A multifoveal, extrafoveal GA with peripapillary atrophy (*arrow*). The vision transformer models FPN_mViT and UNet_mViT agree the most with ground truth. Almost all other models have difficulty separating peripapillary atrophy from GA.

Additionally, a subjective assessment was incorporated by three masked graders (CF, JB, and CP) who visually evaluated and rated the performance of each image in the external testing set. For each image, a panel of 13 segmentation masks were labeled 1-13 without indication of ground truth (human annotation) along with each of the 12 architecture annotations. The graders used a four-level scoring system to assess their agreement with the segmentation mask. A score of 1 was defined as excellent segmentation with no edits to the masks needed, 2 was defined as good with only minor edits. A score of 3 was defined as fair but with some critical errors that needed to be edited (e.g., foveal involvement or peripapillary involvement) (Fig. 2B). Finally, a score of 4 was defined as poor with the segmentation needing to be mostly redone. Images were evenly split among the three graders with a subset graded by all three to ascertain reproducibility. The agreement among the graders was 67%, with most disagreements in the excellent/good scores.

## Results

The detailed demographic characteristics of the training and test datasets are presented in Table 1. A visual representation of predicted GA masks by all 12 models on a single training dataset is shown in Figures 2A and B. The mean area of GA for AREDS2 training dataset was 6.65 mm^2^ (SD 6.30, range 0.10–36.30) and for the testing (external validation) was 9.79 mm^2^ (SD = 5.60, range 0.4–24.3).

**Table 1.** GA Characteristics From Autofluorescence Images in the Internal Cross-Validation and External Validation/Test Datasets

	Training (Cross-Validation)	Testing (External Validation)
Clinical Trial	AREDS2	GSK BAM114341
No. of images (participants)	601 (271)	156 (100)
GA inclusion criteria	Minimum size 0.05 mm^2^, No maximum size	GA area from color photographs 1.9 to 17 mm^2^
Area of GA (mm^2^), mean (SD)	6.65 (6.30)	9.79 (5.60)
Subfoveal GA (%)	48	63
Multifocal GA (%)	18	21

### Model Performance on Dice Coefficients

**Figure 3.**
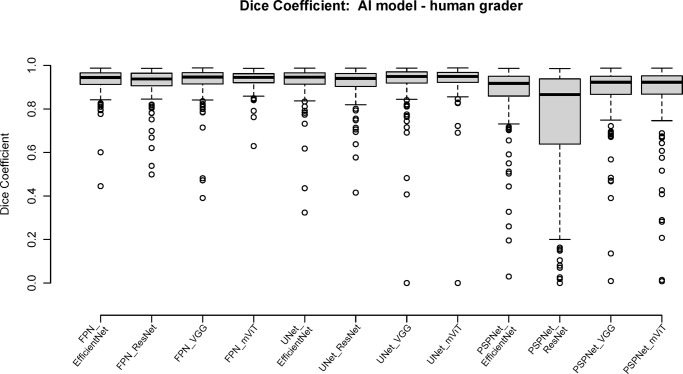
Box plot showing the distribution of dice coefficients across the 12 models in the test dataset. A dice score closer to 1 indicates excellent agreement in spatial overlap of segmented pixels between AI and grader. All models have a Dice score >0.8. The mVit with either FPN or UNet have the lowest variability in dice coefficients.

**Figure 4.**
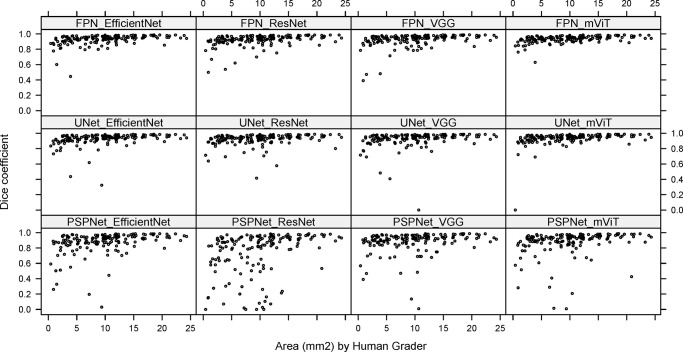
Scatterplots displaying the distribution of dice coefficients from all 12 models for GA segmentation of variable sizes in the test dataset. Overall, dice coefficients are generally better for larger areas of GA in comparison to smaller areas indicating that the models perform better with larger areas.

In the AREDS2 dataset, mean CV dice coefficient from all 12 models was in the range of 0.827 to 0.928, with lowest score obtained on PSP_ResNet and highest score demonstrated by FPN_mViT and UNet_mViT models, as shown in Table 2 and in Supplementary Figure S1 of Supplementary Material. Models showed similar performance in GSK external validation (EV) dataset, with dice ranging between 0.877 to 0.939 as shown in Table 3 and Figure 3. All models showed moderately high performance on precision (CV range 0.877–0.940, EV range 0.930–0.967) and recall (CV range 0.877–0.940, EV range 0.930–0.967) as shown in Supplementary Table S1. Performance of all 12 models on test dataset is shown in Figure 4 displaying the distribution of Dice Coefficient based on GA area. All models unanimously depicted higher dice score and better segmentation for large-sized GAs, while the models showed a more variable performance on smaller areas of GA. Among all 12 models, PSPNet demonstrated the highest variation in dice scores across differently sized GAs, whereas UNet_mViT and FPN_mViT showed more consistent performance across different GA sizes.

**Table 2.** Performance Metrics for the 12 AI Models Assessing GA Area in the Cross-Validation AREDS2 Dataset

Architecture	Mean Area (SD) With AI (mm^2^)	Mean Difference (mm^2^) Between AI and Human Measurement (95% CI)	Correlation Coefficient (*R*)	Dice Coefficient
FPN_EfficientNet	6.58 (6.14)	−0.08 (−1.35, 1.19)	0.99	0.924
FPN_ResNet	6.42 (5.94)	−0.24 (−2.75, 2.27)	0.98	0.919
FPN_VGG	6.59 (6.10)	−0.06 (−2.73, 2.61)	0.93	0.923
FPN_mViT	6.56 (6.18)	−0.09 (−1.99, 1.81)	0.99	0.928
UNet_EfficientNet	6.52 (6.23)	−0.14 (−1.86, 1.58)	0.99	0.924
UNet_ResNet	6.81 (6.32)	0.16 (−3.33, 3.65)	0.96	0.908
UNet_VGG	6.50 (6.15)	−0.16 (−2.45, 2.13)	0.98	0.918
UNet_mViT	6.66 (6.34)	0.00 (−1.55, 1.55)	0.99	0.928
PSPNet_EfficientNet	6.46 (6.23)	−0.20 (−4.73, 4.33)	0.93	0.878
PSPNet_ResNet	5.92 (5.61)	−0.73 (−5.75, 4.29)	0.91	0.827
PSPNet_VGG	6.09 (5.55)	−0.56 (−4.34, 3.22)	0.95	0.887
PSPNet_mViT	6.20 (5.69)	−0.46 (−4.16, 3.24)	0.96	0.880

**Table 3.** Performance Metrics for the 12 AI Models Assessing GA Area in the GSK External Validation/Test Dataset

Architecture	Mean Area (SD) With AI (mm^2^)	Mean Difference (mm^2^) Between AI and Human Measurement (95% CI)	Correlation Coefficient (*R*)	Dice Coefficient
FPN_EfficientNet	9.33 (5.31)	−0.46 (−2.52, 1.60)	0.98	0.931
FPN_ResNet	9.33 (5.38)	−0.46 (−2.62, 1.70)	0.98	0.902
FPN_VGG	9.44 (5.36)	−0.35 (−2.66, 1.96)	0.98	0.934
FPN_mViT	9.24 (5.34)	−0.54 (−1.91, 0.83)	0.99	0.939
UNet_EfficientNet	9.34 (5.36)	−0.45 (−2.82, 1.92)	0.98	0.924
UNet_ResNet	9.04 (5.35)	−0.75 (−3.22, 1.72)	0.97	0.930
UNet_VGG	9.37 (5.50)	−0.41 (−3.08, 2.26)	0.97	0.896
UNet_mViT	9.38 (5.35)	−0.41 (−1.84, 1.02)	0.99	0.938
PSPNet_EfficientNet	9.28 (5.59)	−0.51 (−4.19, 3.17)	0.93	0.890
PSPNet_ResNet	7.40 (5.95)	−2.39 (−8.17, 3.39)	0.87	0.877
PSPNet_VGG	8.74 (5.40)	−1.05 (−4.64, 2.54)	0.95	0.900
PSPNet_mViT	8.80 (5.50)	−0.99 (−5.03, 3.05)	0.98	0.889

### Model Performance on Measurement of GA Area

The mean difference in GA area between ground truth and masks generated by each of the 12 models in the cross-validation dataset ranged from −0.73 (95% CI: −5.75, 4.29) to 0.16 (95% CI: −3.35, 3.65) as shown in Table 2. The lowest mean GA area differences were displayed by FPN_Efficientnet and FPN_mViT models. Among all encoders, ResNet demonstrated the highest area difference between ground truth and predicted GA masks, across all 12 models on training dataset. In comparison, the intergrader assessment of GA area in 47 eyes showed a mean difference of 0.36 (−1.03, 1.75) mm^2^ with a Dice coefficient of 0.99.^25^

**Figure 5.**
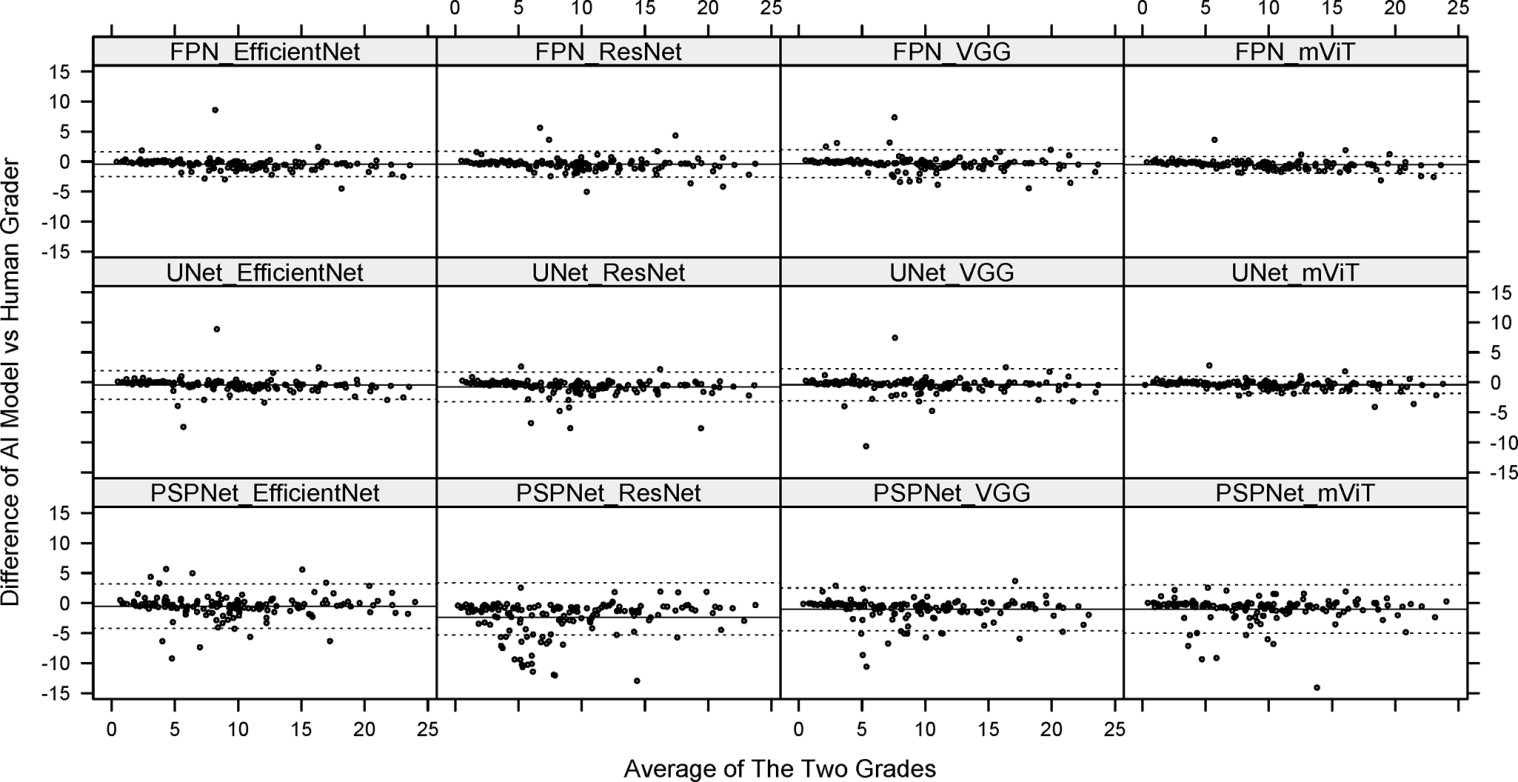
Bland-Altman plots showing the difference between AI-generated segmentation and the ground truth segmentation by human graders of GA areas using the test dataset. All models with the PSPnet architecture have a wide distribution of datapoints indicating weaker agreement between the AI and ground truth. Both FPN and UNet architectures with mViT have the tightest agreement across the range of GA areas.

Similar performance was depicted on the GSK test dataset, with highest variability seen in area measurements from PSPNet architectures whereas mViT based FPN and UNet displayed lowest difference in predicted and ground truth GA area, as shown in Table 3 and Figure 5. Most of the segmentation models compared in this study demonstrated a tendency to overestimate the GA area.

### Model Performance Based on GA Subtypes

We evaluated the performance of all 12 models on GA subtypes, to identify any architecture specific trends in performance for subfoveal vs extrafoveal and unifocal vs multifocal GA. The performance of models on testing dataset shown in Supplementary Figure S2, predominantly indicates larger variability in dice score from PSPNet_ResNet model for smaller sized unifocal GA masks and a more consistent performance by mViT based UNet and FPN models. Similar performance trends were observed for subfoveal and extrafoveal GA, with PSP models showing higher variability for both smaller sized GA of both subtypes, as shown in Supplementary Figure S3. No differences were found in model performance in these subtypes of GA.

### Grader Assessment of Model Performance

**Figure 6.**
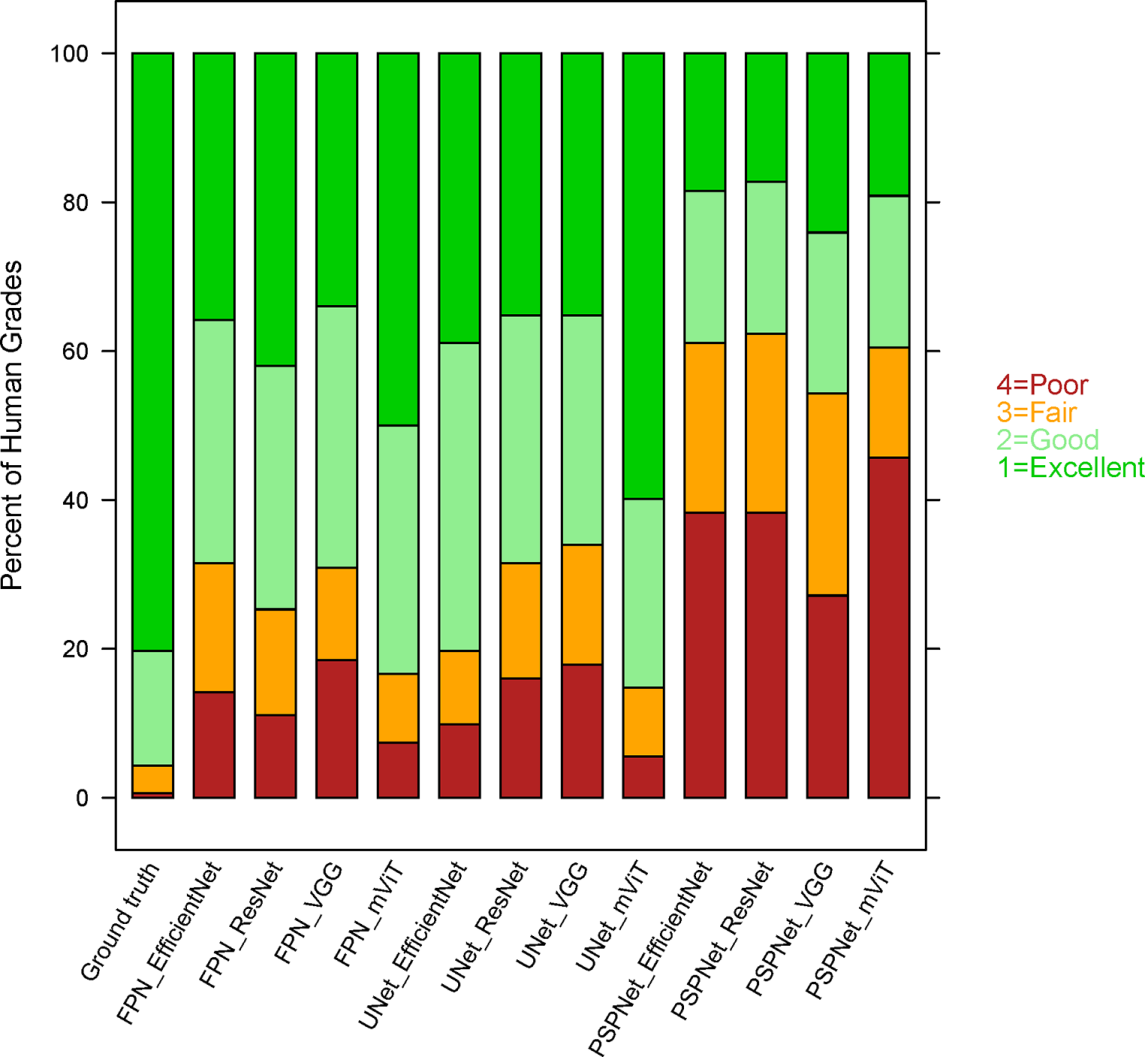
Grader evaluation of AI segmentation model performance on the test dataset using a four-level score. The stacked bar chart displays the percentage distribution of grader scores for each of the 12 models. Graders were presented with 13 distinct masks for scoring for each raw fundus autofluorescence image. The graders were masked to which segmentation was the ground truth as well as the AI architectures.

Anonymized segmentations masks were presented to masked graders for scoring and included 12 AI generated masks and the ground truth. The graders were unaware of which segmentations were done by the human grader nor were they aware of which architecture was used. The ground truth received a score of excellent or good in >95% of images (Fig. 6). The vision transformers (mViT) with UNet and FPN framework received the highest scores among AI generated masks with >75% in the excellent or good category. All other FPN and UNet based architectures ranged around 70% for excellent or good category whereas all PSPNet architectures ranged around 40%.

## Discussion

In this study, we performed a comparative analysis of various AI architectures to understand the implications of architecture variability in GA segmentation. Use of 12 architectures consisting of four encoders and three decoders allowed us to systematically compare variability of combinations. Model performance was compared using Dice coefficient, difference in area between ground truth and AI mask, and a subjective assessment of the GA mask by expert graders. These metrics were evaluated for both the AREDS2 cross-validation dataset and the GSK test dataset. The results show that all 12 architectures have comparable metrics with high performance overall with a Dice score >0.8 across all models. Within the 12 models, the vision transformers encoder with a UNet or FPN architecture performed the best with dice coefficient of 0.93 and least variability on area differences, and >75% GA predictions scored as good or excellent by graders. In contrast the PSPNet architecture with all encoder combinations had the lowest performance with a dice coefficient of <0.9, highest variability in area differences and <40% scoring good or excellent by graders.

There is limited research in effect of architectures on retinal imaging.^26^^,^^27^ Kugelman et al.^26^ evaluated various UNet architectures for segmenting optical OCT scans and found comparable performance across all architectures. Most studies evaluating deep learning for segmenting GA on two-dimensional autofluorescence images have used UNet architecture.^10^^,^^28^ UNet and FPN architectures are similar in their internal structure, with the main difference being that FPN makes predictions at multiple resolutions or scales, whereas UNet makes predictions only at the finest resolution. PSPNet architecture, on the other hand, exploits the global context in the image by pooling or aggregating features from different regions of the feature map, using fully connected layers rather than a skip connection between encoder and decoder framework. This major architectural difference probably leads to inefficient decoding of spatial and global contextual information in the image by PSPNet architecture, thereby limiting its generalization capability in segmenting different phenotypes of GA as depicted in Figure 2.

Both UNet and FPN architectures showed a similar superior segmentation performance compared to PSPNet, with FPN performing slightly better. This could be attributed to the top-down approach used by FPN architecture that enables it to capture multiscale features that can extract fine details and global context in the FAF images, for a robust GA segmentation. In terms of encoders, overall performance is largely similar across CNN based models, with EfficientNet demonstrating higher dice scores, lesser variability compared and better segmentation compared to others. Vision transformers (mViT) showed a consistent performance across the range of area of GA with tight confidence limits compared to CNN models, as shown in Figure 5. This difference could be due to the architectural constraint of CNN based encoders, which are primarily designed to capture local features effectively and therefore may struggle to model long-range dependencies and in capturing global context in the image. This may lead to poor segmentation of distant multifocal GA lesions. There is a possibility of a different trend in relative segmentation performance of encoders depending on the image resolution and model hyperparameters. Considering the vision transformers (mViT) recent emergence as an alternative to conventional CNNs, there is potential for yet undiscovered optimizations that may further enhance its performance.^29^

The Dice coefficient is high (>0.87) for all models; however, the box plots in Figure 3 show outliers across all models, particularly with PSPNet. The Dice Coefficient relies on the intersection of pixels between the two segmentations being compared and the total number of pixels in the ground truth and predictions. When the segmentation area is small, false-positive and false-negative results have a more substantial effect on the Dice Coefficient compared to larger areas. Smaller areas have smaller number of pixels to match on and even if a few mismatches lower the Dice. This is clearly seen in Figure 4 where the Dice is lower for smaller lesions and improves with larger area. Bland-Altman plots and Dice coefficients offer distinct viewpoints for comparison, one offering numerical differences and the other spatial overlap. In a scenario where different lesions of similar area are measured by AI and ground truth, the Bland Altman plots could indicate misleadingly excellent agreement, but Dice coefficients reveal poor performance.

Although statistical metrics provide an insight into model performance, this project used a unique subjective assessment. In clinical trials, GA assessment is usually performed by two graders with a third senior grader reviewing the evaluation and selecting the more accurate one as the definitive version. A similar exercise was completed with 13 unlabeled segmentations presented to the graders (AI predictions and ground truth) for assessment of accuracy. Despite the masking, ground truth segmentations were given an excellent/good score in >95% whereas the best performing vision transformers received similar scores in ∼75% of segmentations. This highlights the importance of using a multifaceted assessment of AI models using real world methods. In addition, other factors such as computational time, the energy required for training and inference (and thus the carbon footprint and real monetary cost), and ease of integration into clinical workflows are also critical to evaluate the model's practical utility. These considerations impact not just the feasibility of deploying a model in a real-world setting but also its environmental and economic sustainability.^30^ Balancing these aspects with performance metrics is essential for a holistic assessment of AI models.

This project was conceived with the hypothesis that certain architectures would perform better with specific phenotypes, such as unifocal versus multifocal GA and extrafoveal vs subfoveal GA. Multifocal GA is more complicated to segment as multiple lesions need to be identified and segmented. Segmentation of extrafoveal GA is complicated as the normal decreased autofluorescence of the fovea in FAF images needs to be differentiated from that of GA. Lack of OCT is a limitation for accurate assessment of foveal involvement in this dataset. As an extension to this hypothesis, our proposal was to selectively invoke specific AI models based on the phenotype presented. However, no significant variance was observed in model performance across different phenotypes such as multifocal/unifocal and subfoveal/extrafoveal, leading us away from the invocation concept. The general trends of performance in the overall dataset reflected in various phenotypes also, with vision transformers encoders with UNet and FPN architectures performing the best across all phenotypes. We then considered an ensemble model incorporating the highest-performing architectures. The ensemble model generated predictions by averaging the predictions from the top 4 models- FPN_EfficientNet, FPN_mViT, UNet_EfficientNet and UNet_mViT. The performance improvement of the ensemble model was marginal compared to the best performing framework, with a dice score of 0.931 on the validation split from AERDS2 dataset, and dice score of 0.925 on the GSK test dataset. The incremental gains did not convincingly outweigh the added complexity.

This study's strengths are in the comprehensive comparison of 12 architectures in a large phenotypically diverse dataset such as AREDS2 and external validation in a clinical trial dataset. The AREDS2 study included eyes with intermediate AMD in one of or both eyes and as such did not have an area cutoff, including both prevalent and incident GA. As such the training dataset had a large area range from 0.1–36.3 mm^2^. One of the challenges with real world implementation of AI models is degradation of model performance, primarily because of selective nature of the training data. Models trained on selected data may not perform well in the real world. In contrast, the model in this project was trained on nearly real-world representative images with diverse presentations of GA from AREDS2 dataset and tested on a clinical trial selective data.^25^ Ground truth was established by experienced readers and performance metrics used grader workflow. To maintain comparison between the various architectures, we controlled various hyperparameters such as the optimizer, learning rate and general model size (number of parameters). Standardizing hyperparameters might not have caused each model to perform optimally, and the limited improvement from the ensemble approach suggests a complexity-performance trade-off.

This research provides a careful assessment of AI architectures for GA segmentation and concludes that vision transformers offer the best performance for this specific task and dataset. It underscores the need for ongoing assessments of emerging AI technologies to optimize performance on the metrics of interest, particularly for slow growing lesions such as GA, where precise measurements are critical. It is important to explore and assess a variety of architectures, to identify the most effective approach for the specific needs and challenges of medical imaging. Selecting and testing for the appropriate AI architecture is an important part of model development and should be aligned with the project's distinct objectives.

## Supplementary Material

Supplement 1

Supplement 2

Supplement 3

Supplement 4

Supplement 5
