# Supplementary material for: Quantifying Geographic Atrophy in Age-Related Macular Degeneration: A Comparative Analysis Across 12 Deep Learning Models
Source: Invest Ophthalmol Vis Sci. 2024 Jul 24;65(8):42. doi: 10.1167/iovs.65.8.42 (PMC11271806; doi:10.1167/iovs.65.8.42)
Supplement: Supplement 1 [file iovs-65-8-42_s001.pdf]

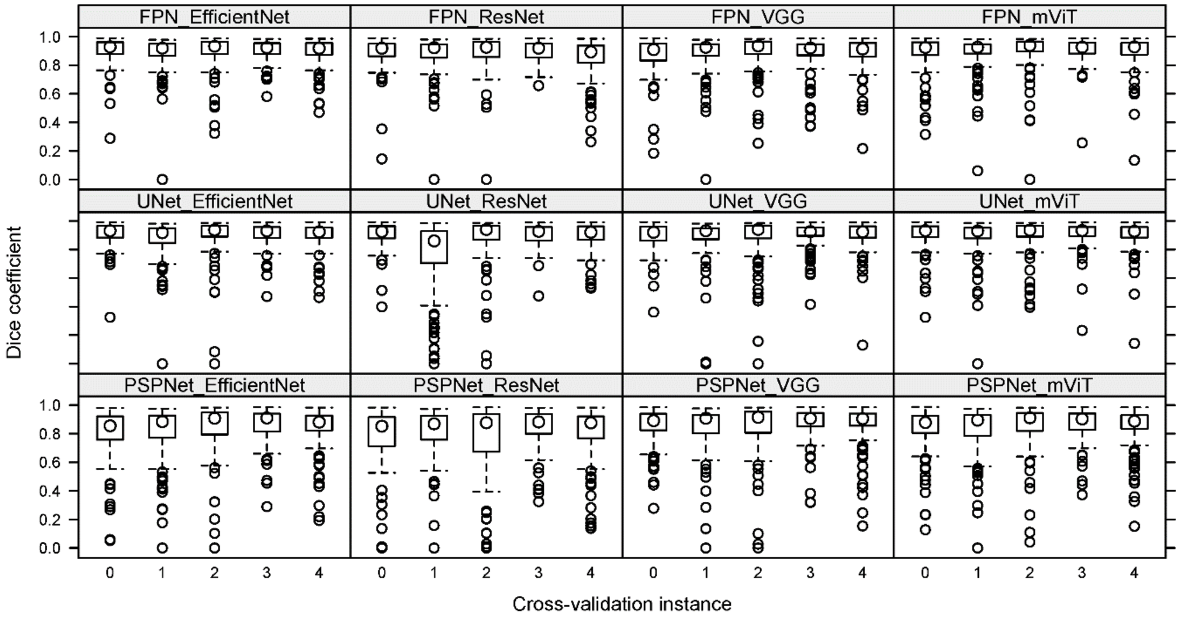

Supplementary Figure 1: Distribution of dice coefficients across the 12 models in the AREDS 5-fold cross validation dataset. A dice score closer to 1 indicates excellent agreement in spatial overlap of segmented pixels between AI and grader. Variability is the lowest on FPN and UNet architectures and is the largest with PSPNet.
