# Supplementary material for: Quantifying Geographic Atrophy in Age-Related Macular Degeneration: A Comparative Analysis Across 12 Deep Learning Models
Source: Invest Ophthalmol Vis Sci. 2024 Jul 24;65(8):42. doi: 10.1167/iovs.65.8.42 (PMC11271806; doi:10.1167/iovs.65.8.42)
Supplement: Supplement 3 [file iovs-65-8-42_s003.pdf]

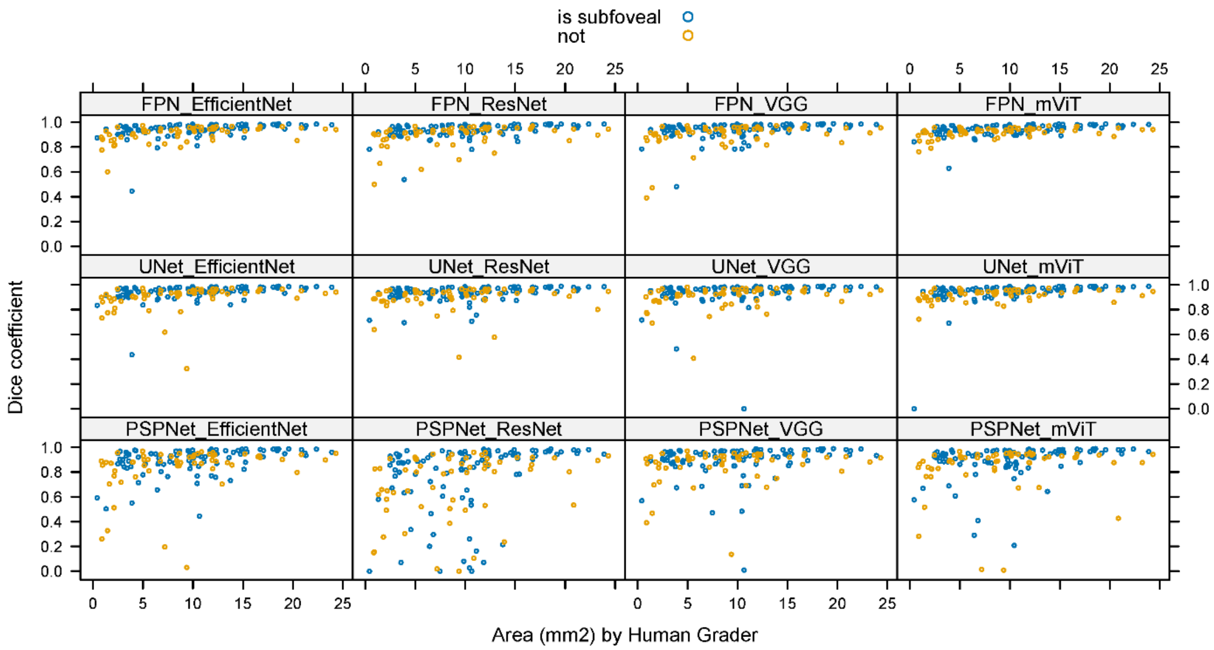

Supplementary Figure 3: Scatterplots indicating distribution of dice coefficients from all 12 models for subfoveal (blue) vs extrafoveal (yellow) geographic atrophy in the GlaxoSmithKline test dataset.
