# Supplementary material for: Quantifying Geographic Atrophy in Age-Related Macular Degeneration: A Comparative Analysis Across 12 Deep Learning Models
Source: Invest Ophthalmol Vis Sci. 2024 Jul 24;65(8):42. doi: 10.1167/iovs.65.8.42 (PMC11271806; doi:10.1167/iovs.65.8.42)
Supplement: Supplement 4 [file iovs-65-8-42_s004.pdf]

Supplementary Table 1: Additional performance metrics for the 12 AI models assessing geographic atrophy area in the cross validation (CV) and external validation (EV) dataset.

Architecture	CV dice	CV jaccard	CV precision	CV recall	EV dice	EV jaccard	EV precision	EV recall
FPN-EfficientNet	0.924	0.925	0.933	0.917	0.931	0.930	0.960	0.908
FPN-ResNet	0.919	0.921	0.925	0.915	0.902	0.908	0.951	0.871
FPN-VGG	0.923	0.924	0.930	0.919	0.934	0.932	0.967	0.906
FPN-mViT	0.928	0.929	0.930	0.929	0.939	0.937	0.967	0.915
UNet-EfficientNet	0.924	0.925	0.939	0.914	0.924	0.924	0.903	0.954
UNet-ResNet	0.908	0.912	0.920	0.903	0.930	0.929	0.962	0.904
UNet-VGG	0.918	0.920	0.940	0.900	0.896	0.900	0.966	0.846
UNet-mViT	0.928	0.928	0.928	0.930	0.938	0.937	0.961	0.919
PSPNet-EfficientNet	0.878	0.885	0.891	0.873	0.890	0.895	0.930	0.866
PSPNet-ResNet	0.827	0.848	0.877	0.805	0.877	0.886	0.953	0.827
PSPNet-VGG	0.887	0.894	0.920	0.864	0.900	0.904	0.951	0.865
PSPNet-mViT	0.880	0.888	0.918	0.852	0.889	0.898	0.936	0.861
