# Supplementary material for: Quantifying Geographic Atrophy in Age-Related Macular Degeneration: A Comparative Analysis Across 12 Deep Learning Models
Source: Invest Ophthalmol Vis Sci. 2024 Jul 24;65(8):42. doi: 10.1167/iovs.65.8.42 (PMC11271806; doi:10.1167/iovs.65.8.42)
Supplement: Supplement 5 [file iovs-65-8-42_s005.pdf]

**Supplementary Material:**

Formulation of performance metrices that were evaluated to assess the segmentation performance of all 12 models is as follows:

$$\text{Dice coefficient} = 2*TP/(2*TP + FN + FP)$$

$$\text{Jaccard index} = TP/ (TP + FN + FP)$$

$$\text{Precision} = TP /( TP + FP)$$

$$\text{Recall or Sensitivity} = TP / (TP + FN)$$

$$\text{Specificity} = TN/(TN+FP)$$

Where TP and FP represent number of true and false positive, whereas TN and FN indicate number of true and false negatives, respectively.
